# An Evaluation of the Safety of Half-Dose Direct Oral Anticoagulants Following Total Joint Arthroplasty: A Pilot Study

**DOI:** 10.7759/cureus.72283

**Published:** 2024-10-24

**Authors:** Harrison A Patrizio, Rex W Lutz, Stephanie A Kwan, Adam Lencer, Gregory K Deirmengian

**Affiliations:** 1 Clinical Education and Assessment Center, Rowan-Virtua School of Osteopathic Medicine, Stratford, USA; 2 Orthopaedic Surgery, Jefferson Health New Jersey, Stratford, USA; 3 Orthopaedic Surgery, Rothman Orthopaedic Institute, Philadelphia, USA

**Keywords:** complication of treatment, therapeutic anticoagulation, total hip arthroplasty (tha), total joint arthroplasty, total knee replacement (tkr)

## Abstract

Background

Total joint arthroplasty (TJA) patients on preoperative anticoagulation therapy present a challenge for adult reconstruction surgeons. The goal in managing such patients is to limit bleeding complications associated with administering the medications while preventing medical complications from withholding them. At our institution, we began a protocol in 2017 that utilizes a half-dose direct oral anticoagulant (DOAC) regimen for one week followed by resuming of the full-dose regimen in select patients who underwent TJA. This study investigated the 90-day safety profile associated with this protocol compared to previous literature.

Methodology

A retrospective review of 898 patients from a single institution was conducted including all patients receiving a half-dose DOAC protocol for one week followed by resuming of the full-dose regimen after total knee and total hip arthroplasty between 2017 and 2022. Data were collected on patient demographics, type of surgery, and DOAC dosage. Ninety-day complications were collected and separated into reduced dose complications of DOAC (such as cerebrovascular accidents (CVAs) or venothrombotic events (VTEe)) and DOAC therapy-related complications (including wound and bleeding complications).

Results

In the total hip arthroplasty (THA) subgroup (n = 396, 44.10%), there were four major VTE occurrences, aligning with the range seen in historical protocols. Deep vein thrombosis (DVT) and pulmonary embolism (PE) incidents were three and one, respectively, both within the historical range. In the total knee arthroplasty (TKA) subgroup of 502 (55.90%) patients, there were seven major VTE events, with five DVTs and two PEs, also aligning with historical ranges. Medical complications in the THA group included no CVA events and one myocardial infarction, with the latter slightly higher than the historical range. In the TKA group, there were two medical complications, both being CVAs. Regarding bleeding complications, THA patients showed four major bleeding incidents, two blood transfusions, and two hemorrhages, with these numbers comparable to or lower than historical ranges. There were seven minor bleeding events. For TKA, there were two major bleeding events, two blood transfusions, no hemorrhages, and five minor bleeding events. Wound complications in THA included five surgery-related complications, two cases of wound dehiscence, and three infections. TKA patients experienced 17 surgery-related complications, six cases of wound dehiscence, and 11 infections.

Conclusions

This study suggests that the half-dose DOAC protocol in patients undergoing TJA is non-inferior to historical full-dose DOAC protocols warranting further investigation to generalize across broader populations.

## Introduction

While total joint arthroplasty (TJA) offers pain relief, functional restoration, and a high rate of favorable outcomes, it is not without risk of complications [1-3]. Possible medical complications include medical events such as venous thromboembolic events (VTEs) and stroke. Patients with a risk of perioperative clotting events often require an individualized approach to postoperative anticoagulation management. Direct oral anticoagulants (DOACs) have revolutionized VTE prophylaxis with their safety and efficacy profiles proving favorable over older anticoagulants such as warfarin [4]. However, questions remain regarding their optimal dosing, especially in postoperative TJA patients [5].

If utilized as primary VTE pharmacological prophylaxis, patients undergoing TJA are often given either rivaroxaban 10 mg daily or apixaban 2.5 mg twice daily [6,7]. However, those already taking DOACs often continue their higher-dose anticoagulation therapy post-TJA [8]. This approach is intended to counter the increased thrombotic risks associated with both the surgery and their pre-existing conditions, with typical dosages of rivaroxaban 20 mg daily and apixaban 5 mg twice daily [6,7]. However, the perioperative period presents a unique challenge in balancing the postoperative bleeding risk with the thromboembolic risk.

Bleeding events can lead to serious complications such as hematoma formation, wound drainage, and subsequent infections and may require additional surgeries, potentially offsetting the benefits of aggressive anticoagulation [9]. With that, guidelines from the American College of Cardiology recommend that patients with atrial fibrillation on DOACs should cease their medication one day before low-bleeding-risk procedures and two days before high-bleeding-risk procedures, followed by resuming DOACs 24 hours after low-bleeding-risk procedures and two to three days following high-bleeding-risk procedures [8]. Additionally, the American Society of Regional Anesthesia (ASRA) recommends that DOACs be paused 24 hours before low-risk procedures and 72 hours before high-risk surgeries, with DOACs resumed after 24 hours postoperatively with consideration of neuraxial anesthesia [10].

Given the bleeding risk, simply resuming the therapeutic DOAC dose in the postoperative period may not be the best means of balancing the risks of postoperative thromboembolic and bleeding complications. In 2017, our institution initiated a protocol that employs a half-dose DOAC regimen, rivaroxaban 10 mg daily and apixaban 2.5 mg twice daily, for hypercoagulable patients for the first week post-TJA, followed by the patient’s preoperative dosage. This pilot study aims to investigate the 90-day safety profile associated with this protocol.

## Materials and methods

In 2017, our institution initiated the following protocol for all patients taking rivaroxaban 20 mg daily or apixaban 5 mg twice daily at baseline for a history of hypercoagulability or atrial fibrillation. Per ASRA guidelines, all patients discontinued the DOAC 72 hours preoperatively in preparation for neuraxial anesthesia. Preoperative anticoagulation bridges were not prescribed. On postoperative day one, all patients were administered half-dose DOAC for seven days, after which their full-dose preoperative regimen resumed. Following institutional review board approval, our research database was used to retrospectively collect all patients from 2017 to 2022 who had undergone total knee arthroplasty (TKA) or total hip arthroplasty (THA) and followed the above protocol. All charts were reviewed to confirm that patients followed the described protocol. Patients were excluded if they deviated from the protocol, which included failing to resume the prescribed half-dose DOAC postoperatively, skipping doses, or adjusting doses outside the protocol. Additionally, patients with incomplete or missing chart data were excluded from the analysis.

Data collection included patient demographics, type of surgery, and DOAC dosage. The specific DOAC used, apixaban or rivaroxaban, and the DOAC indication, such as atrial fibrillation or VTE, were also recorded. Ninety-day mortality, postoperative bleeding events, and prothrombotic complications were collected. Postoperative events were defined as events following surgery. Primary outcomes included postoperative clotting and bleeding complications, with all other data being secondary outcomes. Prothrombotic complications included deep venous thrombosis (DVT), pulmonary embolism (PE), cerebrovascular accident (CVA), and myocardial infarction (MI). Bleeding complications included major bleeding defined as bleeding leading to blood transfusion or hemorrhage, and minor bleeding encompassing all other bleeding such as gastrointestinal bleeding, hematoma, and hematuria. Wound complications such as wound dehiscence and wound infection were also collected. These data were used to determine the safety profile of our DOAC protocol and compare it to historical controls of previous DOAC studies in the current literature.

Historical controls were selected for their relevance to our cohort and study quality. Due to limited current DOAC research, we prioritized high-quality studies with detailed reporting on thromboembolic and bleeding complications in TJA patients receiving full-dose DOAC prophylaxis. Preference was given to studies that closely matched our cohort in demographics and protocols. Studies with incomplete outcome reporting were excluded.

## Results

In this study, 898 patients were included in the cohort for analysis. The cohort consisted of 44.90% males (n = 403) and 55.10% females (n = 495), receiving apixaban (71.60%, n = 643) or rivaroxaban (28.40%, n = 255). All patients underwent either primary THA (44.10%, n = 396) or TKA (55.90%, n = 502) (Table 1).

**Table 1:** Demographic data of the cohort of patients included in our study. THA: total hip arthroplasty; TKA: total knee arthroplasty; BMI: body mass index; DOAC: direct oral anticoagulant; ASA: American Society of Anesthesiologists; CCI: Charlson Comorbidity Index

Demographics	Total (N = 898)	THA (N = 396, 44.10%)	TKA (N = 502, 55.90%)
Age (years) (mean ± SD)	70 ± 10	70 ± 10	70 ± 10
Sex			
Male (N, %)	403 ± 45	194 ± 49	209 ± 42
Female (N, %)	494 ± 55	201 ± 51	293 ± 58
BMI (kg/m^2^) (mean ± SD)	30.8 ± 5.5	30.2 ± 5.6	31.4 ± 5.45
DOAC type			
Apixaban (N, %)	643 ± 71.6	272 ± 68.69	371 ± 73.9
Rivaroxaban (N, %)	255 ± 28.4	124 ± 31.31	131 ± 26.1
Laterality			
Left (N, %)	407 ± 45.3	185 ± 46.7	222 ± 44.2
Right (N, %)	455 ± 50.7	204 ± 51.5	251 ± 50.0
Bilateral (N, %)	36 ± 4.0	7 ± 1.8	29 ± 5.8
ASA (mean ± SD)	2.8 ± 0.7	2.76 ± 0.73	2.73 ± 0.71
CCI (mean ± SD)	4.7 ± 1.9	4.7 ± 1.92	4.67 ± 1.84

DOACs were primarily indicated for atrial fibrillation (40.90%, n = 367) or a history of VTE (40.30%, n = 362). See Table 2 for a full list of DOAC indications.

**Table 2:** Direct oral anticoagulation agent indications for the cohort of patients included in our study. THA: total hip arthroplasty; TKA: total knee arthroplasty; VTE: venous thromboembolic event; DVT: deep vein thrombosis; PE: pulmonary embolism

Indications	Total (N = 898)	THA (N = 396)	TKA (N = 502)
Atrial fibrillation (N, %)	367 (40.90)	173.0 (26.90)	194.0 (38.60)
VTE history (N, %)	362 (40.30)	124 (19.20)	238 (47.40)
DVT history (N, %)	191 (21.30)	85.0 (13.20)	106.0 (21.10)
PE history (N, %)	98 (10.90)	39.0 (6.10)	59.0 (11.80)
Stroke history (N, %)	25 (2.80)	8.0 (1.20)	17.0 (32.70)
Myocardial infarction history (N, %)	8 (0.90)	5.0 (0.80)	3.0 (0.60)
Other coagulation disorders (N, %) (factor V Leiden, factor XI deficiency, lupus anticoagulant disorder, polycythemia vera, G6PD deficiency, homocystinuria, prothrombin gene mutation, antiphospholipid antibody syndrome)	9 (1.00)	3 (0.50)	6 (1.20)
Other vascular disorders (N, %) (valvular heart disease, atherosclerosis of aorta, bilateral carotid disease, coronary artery disease)	19 (2.10)	8 (1.20)	11 (2.20)
Cancer (N, %) (breast, lung, prostate, colon, etc.)	35 (3.90)	17 (2.60)	18 (3.60)
Physician decision (N, %)	73 (8.10)	35 (5.40)	38 (7.60)

No mortalities were observed within the studied patient population within the follow-up period. In the THA subgroup, major VTE occurrences in the half-dose DOAC protocol were 1.01%. DVT incidents were 0.76% and PE incidents were 0.25%. In the TKA group, major VTE was noted at 1.39%, DVT at 1.00%, and PE at 0.40% (Tables 3, 4).

**Table 3:** Venous thromboembolic complications for THA in study patients compared to historical controls. THA: total hip arthroplasty; VTE: venous thromboembolic event; DVT: deep vein thrombosis; PE: pulmonary embolism

	Half dose (present study), N = 396		Piple et al. (2023), N = 42,469 [11]		Glassberg et al. (2019), N = 15,631 [12]		Colwell et al. (2014), N = 6,699 [13]	
Complications	Events	%	Events	%	Events	%	Events	%
Major VTE	4	1.01	212	0.50	310	1.98	48	0.72
DVT	3	0.76	134	0.32	141	0.90	40	0.60
PE	1	0.25	78	0.18	169	1.08	8	0.12

**Table 4:** Venous thromboembolic complications for TKA in study patients compared to historical controls. TKA: total knee arthroplasty; VTE: venous thromboembolic event; DVT: deep vein thrombosis; PE: pulmonary embolism

	Half dose (present study), N = 502		Piple et al. (2023), N = 86,721 [11]		Colwell et al. (2016), N = 5,351 [13]		Kulshrestha et al. (2013), N = 450 [14]	
Complications	Events	%	Events	%	Events	%	Events	%
Major VTE	7	1.39	728	0.84	71	1.33	10	2.22
DVT	5	1.00	497	0.57	68	1.27	8	1.78
PE	2	0.40	231	0.27	3	0.06	2	0.44

Among all patients who experienced VTE complications, 10 (90.90%) patients had a prior VTE history, and one (9.10%) patient had a history of atrial fibrillation.

The overall rate of CVA and MI complications in the THA group for our half-dose protocol was 0.25%. The incidence of CVA was 0.00% and MI was 0.25%. The TKA group showed a complication rate of 0.40% for CVA and 0.00% for MI (Tables 5, 6).

**Table 5:** CVA and MI complications for THA in study patients compared to historical controls. THA: total hip arthroplasty; CVA: cerebrovascular accident; MI: myocardial infarction

	Half dose (present study), N = 396		Piple et al. (2023), N = 42,469 [11]		Huang et al. (2019), N = 192 [15]	
Complications	Events	%	Events	%	Events	%
Total	1	0.25	72	0.17	0	0.00
CVA	0	0.00	29	0.07	0	0.00
MI	1	0.25	43	0.10	0	0.00

**Table 6:** CVA and MI complications for TKA in study patients compared to historical controls. TKA: total knee arthroplasty; CVA: cerebrovascular accident; MI: myocardial infarction

	Half dose (present study), N = 502		Piple et al. (2023), N = 86,721 [11]		Lassen et al. (2008), N = 1,220 [16]	
Complications	Events	%	Events	%	Events	%
Total	2	0.40	145	0.17	4	0.33
CVA	2	0.40	72	0.08	3	0.25
MI	0	0.00	73	0.08	1	0.08

Notably, both cases of CVA occurred in patients with a previous history of VTE, while neither of these patients had a history of atrial fibrillation.

Regarding bleeding complications, the rate of major bleeding incidents in THA patients in the half-dose protocol was 1.01%. Blood transfusions were at 0.51% and hemorrhage at 0.51%. The rate of minor bleeding events was 1.77%. For TKA, the rate of major bleeding events was 0.40%, blood transfusions 0.40%, hemorrhage 0.00%, and minor bleeding 1.00% (Tables 7, 8).

**Table 7:** Bleeding complications for THA in study patients compared to historical controls. THA: total hip arthroplasty

	Half dose (present study), N = 396		Piple et al. (2023), N = 42,469 [11]		Huang et al. (2019), N = 192 [15]		Levitan et al. (2014), N = 10,000 [17]	
Complications	Events	%	Events	%	Events	%	Events	%
Major bleeding	4	1.01	1,372	3.23	1	0.52	39	0.39
Blood transfusions	2	0.51	1,296	3.05	0	0.00	21	0.21
Hemorrhage	2	0.51	76	0.18	1	0.52	18	0.18
Minor bleeding	7	1.77	126	0.30	2	1.04	334	3.34

**Table 8:** Bleeding complications for TKA in study patients compared to historical controls. TKA: total knee arthroplasty

	Half dose (present study), N = 502		Piple et al. (2023), N = 86,721 [11]		Lassen et al. (2008), N = 1,220 [16]		Levitan et al (2014), N = 10,000 [17]	
Complications	Events	%	Events	%	Events	%	Events	%
Major bleeding	2	0.40	1,451	1.67	2	0.16	146	1.46
Blood transfusions	2	0.40	1,267	1.46	1	0.08	75	0.75
Hemorrhage	0	0.00	184	0.21	1	0.08	71	0.71
Minor bleeding	5	1.00	165	0.19	33	2.70	278	2.78

Wound complications in the THA group showed surgery-related complications at 1.26% for the half-dose protocol. Wound dehiscence was 0.51% and wound infection 0.76%. In the TKA group, surgery-related complications were 3.39%, wound dehiscence 1.20%, and wound infection 2.19% (Tables 9, 10).

**Table 9:** Wound and infection complications for THA in study patients compared to historical controls. Wilson et al. (2023) [18] included only wound dehiscence data and Pincus et al. (2020) [21] included only infection data, so the two studies were included to provide a full complement of historical data. THA: total hip arthroplasty

	Half Dose (present study), N = 396		Wilson et al. (2023) N = 3,687 [18]		Lee et al. (2015), N = 11,810 [19]		Jewett et al. (2011), N = 800 [20]		Pincus et al. (2020), N = 2,993 [21]	
Complications	Events	%	Events	%	Events	%	Events	%	Events	%
Surgery related	5	1.26	98	2.66	165	1.40	20	2.50	36	1.20
Wound dehiscence	2	0.51	98	2.66	111	0.94	13	1.63		
Wound infection	3	0.76			54	0.46	7	0.88	36	1.20

**Table 10:** Wound and infection complications for TKA in study patients compared to historical controls. TKA: total knee arthroplasty

	Half Dose (present study), N = 502		George et al. (2018), N = 140,199 [22]		Newman et al. (2011), N = 181 [23]		Kim et al. (2017) [24]		
Complications	Events	%	Events	%	Events	%	Events	Total	%
Surgery related	17	3.39	1,401	1.00	7	3.87	21	890	2.36
Wound dehiscence	6	1.20	280	0.20	3	1.66	6	364	1.65
Wound infection	11	2.19	1,121	0.80	4	2.21	15	526	2.85

## Discussion

In this retrospective pilot study, we evaluated the efficacy and safety of a half-dose DOAC regimen in patients who were prescribed these medications preoperatively for various indications, including hypercoagulable conditions, atrial fibrillation, and other factors such as physician discretion based on individualized risk assessments. Typically, these patients require higher DOAC dosages due to an increased risk of clotting and thrombotic events [8]. However, for this study, the dosages were halved, from 20 mg to 10 mg daily for rivaroxaban and from 5 mg to 2.5 mg twice daily for apixaban for one week, after which they resumed the full dose. These half doses are the standard prophylactic doses for patients with hypercoagulable conditions undergoing TJA who are prescribed DOACs for VTE prophylaxis [6,7]. Importantly, bridging therapy with low-molecular-weight heparin was not required [25]. The Perioperative Anticoagulant Use for Surgery Evaluation (PAUSE) trial demonstrated that perioperative interruption of DOACs, including rivaroxaban and apixaban, without bridging, is both safe and effective, with low rates of major bleeding (1.00%) and arterial thromboembolism (0.50%) [25]. This also aligns with findings from the BRIDGE trial, which showed that forgoing bridging in warfarin patients similarly reduced bleeding risk without increasing thromboembolic events [26].

Our findings suggest that this adjusted half-dose regimen, while lower than what is typically prescribed for high-risk patients, matches the efficacy and safety of standard DOAC doses in the general population. This equivalence implies a half-dose DOAC for one week following TJA may be non-inferior to a full-dose DOAC in managing their elevated clotting risk while also limiting bleeding and wound complications. This result is important, as it indicates the potential for using a reduced DOAC dosage to provide effective thromboprophylaxis in patients with a history of hypercoagulability and atrial fibrillation while attempting to minimize bleeding and wound complications.

In assessing thromboprophylaxis efficacy, our findings are particularly noteworthy. The incidences of major VTE, DVT, and PE in both the THA and TKA groups were in line with those observed in historical protocols [11-14]. Despite the heightened thrombotic risk inherent in patients with a hypercoagulable history, the incidences of major VTE were quite low, especially when considering the annual clotting complication rates reported in broader patient populations such as atrial fibrillation [5]. Specifically, the risk of major VTE in THA patients was only 1.01%, significantly lower than the general yearly clotting risk reported in similar patient groups, such as the 1.27% rate among atrial fibrillation patients on DOACs found by Granger et al. [5] Additionally, our rate of VTE is comparable to aspirin prophylaxis rates of approximately 1.30% seen in healthy TJA patients [27]. Furthermore, in the TKA cohort, the incidence of major VTE was slightly higher at 1.39% on the half-dose protocol; however, previous research has shown rates as high as 2.22% in TKA [14].

The safety profile of the half-dose regimen, in particular, postoperative bleeding, is compelling. In our study, the incidence of major bleeding and the need for blood transfusions were comparable to, or lower than, those reported in historical DOAC studies [11,17]. Our bleeding complications also align closely with previously reported rates such as the 1.50% reported by Anderson et al. with the use of aspirin in the general population [28]. This is notable given the hypercoagulable status of many patients in our cohort and the established bleeding complication rate of about 2.13% per year in atrial fibrillation patients on DOACs [5]. The reduced hemorrhage rates in TKA patients highlight the half-dose protocol’s potential to minimize perioperative bleeding complications in high-risk groups [11,16,17]. The strength of this comparison is further emphasized by large-scale studies used for comparison such as Piple et al. (n = 86,721) and Levitan et al. (n = 2,014) [11,17].

The current study also investigated wound-related complications. We found that the incidence of surgical complications such as wound dehiscence and infection was within or below those seen with historical regimens [18-21,23,24]. This is encouraging, as wound healing is a crucial component of TJA recovery, and our results suggest that the half-dose DOAC regimen does not adversely impact this process. However, it is important to note the observed variations in systemic medical complications, such as CVA and MI, under the half-dose protocol, some of which exceeded those in historical regimens [11,15,16]. This raises important considerations about the broader effects of reduced anticoagulation intensity, particularly in a hypercoagulable population. The potential increase in certain complications, such as MI, warrants careful interpretation and further research, especially considering the unique characteristics of our study’s patient population. More comprehensive studies are needed to fully understand the impact of anticoagulation dosing in patients with a hypercoagulable history undergoing TJA.

This study is not without limitations. Its retrospective design and relatively small sample size, combined with a single-center scope, limit the generalizability of our findings. The small sample size may not fully capture the range of complication rates seen in broader populations, as a limited number of events can disproportionately influence the observed complication percentages. Additionally, the lack of a randomized control group, and the absence of a contemporary comparison group, means that our analysis relies solely on historical controls. This reliance on historical data introduces potential selection bias, which could affect the interpretation and perceived effectiveness of the half-dose regimen. To reach more definitive conclusions, future studies should be prospective, with case numbers calculated from the literature in advance, to provide a more robust assessment of the safety and efficacy of the half-dose DOAC protocol. Despite these limitations, our findings consistently align with previous studies, reinforcing the potential efficacy of this regimen. Further research, particularly through prospective studies and randomized controlled trials, will be essential to fully evaluate its effectiveness in a broader and more diverse TJA population as anticoagulation practices continue to evolve.

## Conclusions

This retrospective review supports the hypothesis that a half-dose DOAC protocol in hypercoagulable patients is non-inferior when compared to historical regimens used in previous studies. Further research is warranted to validate these findings across larger and more diverse populations, which will be critical for formulating guidelines that ensure both the efficacy and safety of anticoagulation protocols in TJA.
